# Towards Plant Species Identification in Complex Samples: A Bioinformatics Pipeline for the Identification of Novel Nuclear Barcode Candidates

**DOI:** 10.1371/journal.pone.0147692

**Published:** 2016-01-25

**Authors:** Alexandre Angers-Loustau, Mauro Petrillo, Valentina Paracchini, Dafni M. Kagkli, Patricia E. Rischitor, Antonio Puertas Gallardo, Alex Patak, Maddalena Querci, Joachim Kreysa

**Affiliations:** Molecular Biology and Genomic Unit, Institute for Health and Consumer Protection, Joint Research Center, European Commission, Ispra, Italy; Istituto Biologia e Biotecnologia Agraria IBBA, ITALY

## Abstract

Monitoring of the food chain to fight fraud and protect consumer health relies on the availability of methods to correctly identify the species present in samples, for which DNA barcoding is a promising candidate. The nuclear genome is a rich potential source of barcode targets, but has been relatively unexploited until now. Here, we show the development and use of a bioinformatics pipeline that processes available genome sequences to automatically screen large numbers of input candidates, identifies novel nuclear barcode targets and designs associated primer pairs, according to a specific set of requirements. We applied this pipeline to identify novel barcodes for plant species, a kingdom for which the currently available solutions are known to be insufficient. We tested one of the identified primer pairs and show its capability to correctly identify the plant species in simple and complex samples, validating the output of our approach.

## Introduction

The development of analytical techniques for the correct analysis of plant species in complex samples is of paramount importance for the implementation of various legislations aiming at protecting the rights and the safety of the consumers. In the European Union, frameworks are in place regarding the correct labelling of ingredients (including allergens) [1], adulteration of olive oil [2], wine [3] and pasta [4], and the legal trade definition of cacao [5] and coffee [6], to name just a few.

Because of its relative resilience to food processing, DNA is a target of choice for taxon determination in unknown samples. Although several methods based on the polymerase chain reaction (PCR) have been proposed, these approaches require the development and application of individual assays for each of the taxons to be detected in a sample, thus also requiring *a priori* hypotheses on the species present.

For this reason, recent efforts were made to apply the concepts of DNA barcoding in this context [7][8]. DNA barcoding, first defined in Hebert et al. (2003)[9], involves the amplification, followed by sequencing, of specific DNA sequences. In order to be useful as a DNA barcode, a target needs to satisfy three crucial conditions: firstly, it should be short enough to allow efficient amplification and sequencing; secondly, it should be flanked by regions of high identity across species of interest, to allow the design of broad specificity primers; thirdly, the sequence of the amplified region itself should be sufficiently variable between species to allow assignment of sequenced amplicons to specific species of origin.

For animals, extensive research on DNA barcoding has focused on the mitochondrial cytochrome oxidase 1 (co1) gene (for example, the Barcode Of Life community and associated resources [10]). For plants, however, this region was found inadequate due to the lower nucleotide variability in this region, and recent projects have focused on combinations of plastid markers, such as matK, rbcL, trnH-psbB and others [11], used both for phylogenetic studies [12][13][14] and species identification in unknown mixtures [7][15][16][17][18].

It has however become clear that a complete DNA barcoding solution for plant species identification would likely involve the combination of multiple targets [19]. The nuclear genome, which is orders of magnitude larger and more complex than mitochondrial or plastid genomes, is a good potential source of such targets. Many technologies using DNA fingerprinting for plant species identification target regions of the nuclear genome, detecting, for example, length polymorphism of introns or patterns of transposable elements (for a recent review, see [20]). For barcoding, ribosomal RNA regions (particularly their internal transcribed spacers, ITS)[19][21], as well as low-copy nuclear gene candidates [22][23][24], have been explored as novel target candidates; however, successes have been limited by the stringency of the criteria described above and the complexity of the information to be processed.

In order to solve this problem, we have developed a bioinformatics pipeline designed to automatically screen large numbers of DNA sequences without any *a priori* consideration of their function. Using the currently available whole plant genome sequences (currently for about 140 species) and common bioinformatics tools, the pipeline outputs the sequences of potential primers and associated amplicons that were computed to fulfil the described DNA barcode requirements. We then test one of the output primer pair in a set of Next Generation Sequencing (NGS) experiments and show its capacity to identify the correct plant species present in a set of simple and complex samples.

## Materials and Methods

### Bioinformatics pipeline

A detailed description of the bioinformatics pipeline, and the intermediate results of the instance performed for this article, can be found in the Supporting Information. The plants genome sequences used for the analyses were downloaded from Ensembl (ftp://ftp.ensemblgenomes.org/pub/plants/release-23/). Additionally available genomes were downloaded from Genbank (ftp://ftp.ncbi.nih.gov/genomes/genbank/plant/, accessed 14-10-2014). When more than one version, or subspecies, was available for the same species, the largest file was kept.

A series of scripts were prepared (using the Ruby scripting language) to automatically perform the steps of the pipeline. The scripts made use of the following command line binary tools, installed locally: BLAST version 2.2.29 from NCBI [25], Clustal Omega version 1.2.0 from EMBL [26], HMMER version 3.1 from the Howard Hughes Medical Institute [27], EMBOSS Open Software Suite version 6.5.7 [28], MAFFT version 7.205 [29], Weblogo version 3.3 from UC Berkeley [30], e-PCR version 2.3.12 from NCBI [31], cd-hit-est the CD-HIT package version 4.6.1 from ecoPCR from the Laboratoire d’Ecologie Alpine [32]. The detailed steps of the pipeline are described in the Supporting Information. The scripts performing the various steps are available for download, as Supporting Information S1 Scripts. The seed sequences for the experiments described in the article were obtained from the nr_limes.txt file, downloaded from http://andromeda.rnet.missouri.edu/old/datasets/limes/index.html [33].

### Test materials

A certified reference material (CRM) for maize Bt11 1% (ERM-BF412b) was purchased from the Institute for Reference Materials and Measurements (IRMM), Geel, Belgium; soybean CV127 100% (AOCS 0911-D) was purchased from the American Oil Chemists Society (AOCS), Illinois, USA, and frozen leaves of non-GM rice (Oryza sativa ssp. Japonica, cv. Kaybonnet), previously provided for another study by the International Rice Research Institute (IRRI), Philippines [34] were used. In addition to these materials, a commercial muesli mix and fresh strawberries were purchased from the local market (Italy).

### DNA extraction and quantification

DNA was extracted from 200 mg of starting material; different methods were used based on the matrix from which the DNA was to be extracted: CTAB DNA extraction method was used for soybean (modified from ISO 21571 [35]), NucleoSpin Food kit (Macherey-Nagel GmbH, üren, Germany) for maize, and Biotecon foodproof Sample Preparation Kit III (Biotecon Diagnostics GmbH, Potsdam, Germany) for the muesli mix and strawberries. Rice DNA was extracted from leaves as described elsewhere [34]. The integrity of the extracted genomic DNA was controlled by agarose gel electrophoresis, followed by DNA quantification using Quant-iT PicoGreen dsDNA Assay kit (Life Technologies, Molecular Probes, Eugene, Oregon, USA) and a Bio-Rad VersaFluor fluorometer.

### PCR reactions and optimization

DNA samples were prepared to a concentration of 10 ng/*μ*l unless lower amounts were obtained. Optimization of the amplification conditions and template concentrations was achieved before Next Generation Sequencing (NGS) experiments. The primer set used was 23579-aaa-F: 5’-TCCTTCTGGATGTTGTAGTC-3’ and 23579-aaa-R: 5’-AAGATGCAGATCTTCGTGAA-3’ and the expected generated size was 194 bp. For maize, soy and rice, 40 ng of genomic DNA per reaction were used, for muesli 20 ng were used instead, while for strawberries the amount of DNA was as little as 1.5 ng due to the presence of PCR inhibition observed at higher concentrations. PCR amplifications were performed in 50 *μ*l using 1.25 U/reaction of AmpliTaq Gold DNA Polymerase, 1x Buffer II, 500 nM of each primer, 200 *μ*M dNTPs, and MgCl2 (Applied Biosystems, Roche Diagnostics) at 1.5 mM for the maize and soybean material, and at 4.5 mM for the rest of the samples tested. In addition, DMSO solution (Bio-Rad Laboratories, CA, USA) at a final concentration of 2% was added to the reaction. DNA samples were amplified in a GeneAmp PCR System 9700 (ABI, USA), with the following cycling parameters, according to the protocol of the AmpliTaq Gold PCR system: initial denaturation at 95°C for 10 min, followed by 30 cycles of denaturation at 95°C for 30 sec, annealing at 59°C for 30 sec, extension at 72°C for 30 sec, and final extension at 72°C for 7 min. PCR products were analyzed on an agarose gel electrophoresis to verify and confirm the expected size.

### Barcoding experiments

Next generation sequencing (NGS) experiments were performed using GS Junior System (GS Junior System, 454 Life Sciences, Roche Applied Sciences, Basel, Switzerland). Amplicon libraries were prepared using fusion primers for bidirectional sequencing as described in the Amplicon library preparation manual (Roche Applied Sciences, Basel, Switzerland). Each primer consisted in a 5’-portion 25-mer whose sequence was dictated by the requirements of the 454 Sequencing System for hybridizing to the DNA capture beads (Lib-A), and for annealing the emPCR amplification primers and the sequencing primer. In addition, Multiplex Identifiers (MIDs) were added in order to allow inclusion of more than one sample in the same experiment. Two version of each primer, including either MID1 (ACGAGTGCGT) or MID2 (ACGCTCGACA) were synthesised in order to allow the simultaneous analysis of four samples (MID1-1, MID1-2, MID2-1 and MID2-2) in the same experiment. The final sequences were thus as follows:

23579-F-MID1: 5’-CGTATCGCCTCCCTCGCGCCATCAGACGAGTGCGTTCCTTCTGGATGTTGTAGTC-3’,
23579-F-MID2: 5’-CGTATCGCCTCCCTCGCGCCATCAGACGCTCGACATCCTTCTGGATGTTGTAGTC-3’
23579-R-MID1: 5’-CTATGCGCCTTGCCAGCCCGCTCAGACGAGTGCGTAAGATGCAGATCTTCGTGAA-3’
23579-R-MID2: 5’-CTATGCGCCTTGCCAGCCCGCTCAGACGCTCGACAAAGATGCAGATCTTCGTGAA-3’

After amplification, amplicons were purified using AMPure XP beads (Roche Applied Sciences, Basel, Switzerland), quantified fluorometrically (Quant-iT PicoGreen dsDNA Assay kit, Life Technologies, Molecular Probes, Eugene, Oregon, USA), diluted, and pooled to a final concentration of 0.5x 10^6^ molecules/*μ*l. Libraries were checked for their quality, e.g., presence of short fragments before and after the QC PCR, by performing a Quality Control PCR; they were subsequently visualized using Agilent DNA 1000 Chips (Agilent Bioanalyzer, Agilent Technologies, San Diego, USA). Emulsion PCR containing between 0.6 and 0.75 copies per bead (cpd) was recovered using vacuum and the successive enrichment led to an enrichment rate between 10–20%; only 5% of the enriched beads were subsequently loaded on the chip and sequenced in a GS Junior System (Roche Diagnostics). All steps were in accordance with the manufacturers’ instructions.

The original reads files were analysed by coral, an error correction algorithm [36]. The output (FASTA format) was split using fastx_barcode_splitter (from the fastx-toolkit [37]) to isolate the reads from the different samples tested in the same run using the MID sequences. Prior to clustering, the primer sequences were trimmed using cutadapt [38], and the results pooled with all the reference reads in a single FASTA file for each sample. Clustering was performed using cd-hit-est (cd-hit-v4.6.1)[32], at 100% identity across the whole length of the reads and references, using the following options: -n 8 -c 1.0 -M 0 -T 80 -g 1 -d 0 -aL 1 -aS 1. With this operation, three types of clusters were obtained: clusters containing only reference reads, clusters containing only NGS reads, and clusters containing NGS reads and at least one reference read. These latter were the main focus of the subsequent analyses, from which the number of NGS reads and the species of the reference reads were recorded and plotted. The reads taxonomy plots were generated manually using a simple SVG editor.

### Relative quantification

For relative quantification of maize and soy in the same sample, DNA solutions extracted from the pure samples, as described above, were mixed in 25:75, 50:50 and 75:25 weight:weight ratios. The resulting mixes were used for barcoding experiments, starting from the PCR amplification using the MID-linked primers as described in the previous section. After clustering of the reads with the references, the cluster with the highest number of reads linked to a single reference read was kept for each of the two species. The number of reads were then corrected to the C values of soy and maize (1.13pg and 2.73pg, respectively, according to http://data.kew.org/cvalues/).

## Results

### Bioinformatics pipeline

**Fig 1:**
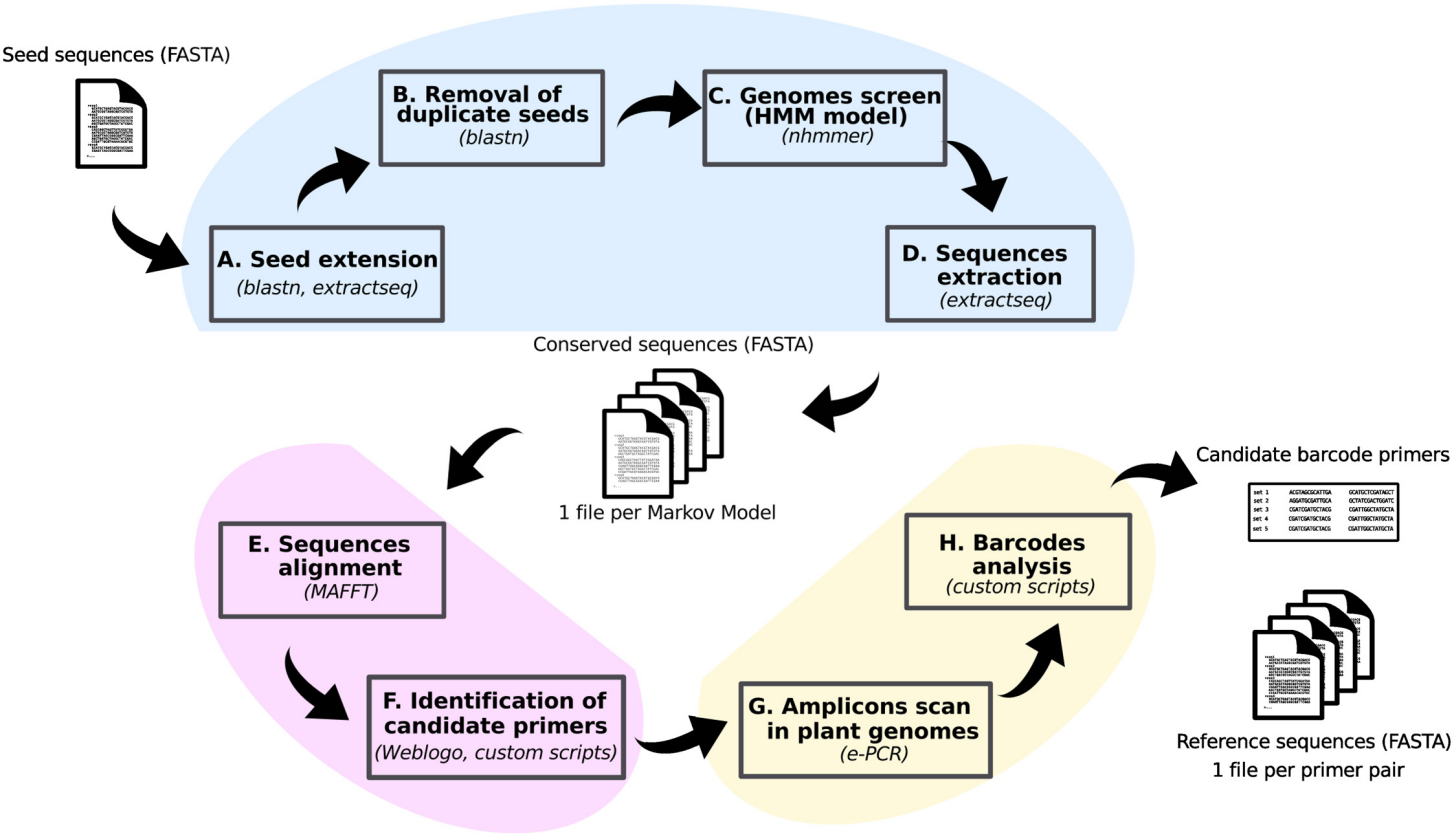
Bioinformatics pipeline for the identification of DNA barcodes in the nuclear genome. Scheme representing the flow of the different steps in the bioinformatics pipeline designed to process input sequences (top left) in order to output potential primer pairs amplifying novel DNA barcoding targets (bottom right). See text and Supporting Information for details.

In order to identify novel genomic DNA barcode candidates, we have developed a bioinformatics pipeline designed to automatically screen large numbers of DNA sequences without any *a priori* consideration of their function. Using the currently available whole plant genome sequences (about 140 species) and common bioinformatics tools, the pipeline outputs the sequences of potential primers and associated amplicons that were computed to fulfil the described DNA barcode requirements. The steps of the pipeline, and the associated tools used, are summarized in Fig 1 and detailed in S1 Text.

Briefly, the first part of the pipeline (Fig 1A–1D) involves the generation, through automated mining of available plant genomes with an input set of candidate DNA sequences, of a high-quality set of sequences significantly conserved in a large number of plant species. The second part of the pipeline (Fig 1E–1F) uses these sets of sequences to identify pairs of 20bp regions of sufficient conservation and separated by 100–350 bases in order to generate barcode primers. The third and final part (Fig 1G–1H) involves the use of these primers to perform *in silico* PCR on each of the whole plant genomes in order to generate a set of predicted amplicon sequences. These primers, and their respective sets of amplicon sequences, represent the main output of the pipeline, and can be mined to select primers adequate for specific purposes.

We have run the pipeline using, as input, a published dataset of 1039 plants complex Long Identical Multispecies Elements (LIMEs) characterised and published by Reneker et al. (2012)[33]. In this work, LIMEs were defined as sequences that are found to be 100% identical and located in syntenic positions in at least two plants genomes and were thus considered to be a good source of potential candidates for barcode regions. In order to experimentally test the validity of the pipeline output, we analysed the resulting primers to select the pair producing at least one unique amplicon in the largest number of species, without particular consideration about the nature of these species or of the level of intra- or inter-species variability in the barcode sequences. With this analysis, the top primer pair candidates are shown in Table 1, together with their corresponding genomic region annotations. Interestingly, a significant number of candidates are in regions coding for rRNA components, similar to previously explored strategies [19][21], which can be seen as an internal control for our pipeline. The best primer with these criteria is 23579-aaa, whose automatically assigned name refers to the first primer pair (aaa) designed in the conserved region derived from the LIME labelled 23579 in Reneker et al. (2012)[33] as a starting seed.

**Table 1:** Primer pairs producing a unique amplicon in the highest number of species, based on the analysis of their genome sequences, using the input set from Reneker et al (2012). The primers sequences can be found in Table C in S1 Text.

Primer pair	Species^1^	Amp size	Annotation	Plants^2^	Invert.^2^	Vert.^2^
23579-aaa	125	194 bp	Ubiquitin family member	615	220	147
24772-aaa	124	218 bp	HSP70 family member	355	3	0
586-aaq	124	320 bp	rRNA	1660	2700	7
25268-aaa	122	206 bp	NAC domain-containing protein	1	0	0
23965-aaa	121	215 bp	NAC domain-containing protein	262	1	0
18638-aqc	121	199 bp	rRNA	1092	140	2
632-aio	116	320 bp	rRNA	1832	2089	2
637-aek	112	294 bp	rRNA	2247	2475	31
24430-aab	109	305 bp	LHCP^3^ complex II subunit B1	292	7	1
24079-aae	107	204 bp	Uncharacterised protein	17	0	0
25227-aay	104	280 bp	RGP protein	36	0	0

^1^From e-PCR scanning of Ensembl and Genbank plant genome sequences

^2^From ecoPCR scanning of the ENA std database (release 123)

^3^Light-harvesting chlorophyll protein

To test the presence of these target regions in other eukaryotes, and in order to enrich the set of reference sequences for barcoding experiments, we mined a local copy of the whole European Nucleotide Archive (ENA) nucleotide database [39] using ecoPCR [40], with each of these primers. The results were then parsed and each species for which a unique amplicon sequence was identified was assigned to the Plants, Vertebrates or Invertebrates divisions using the information from the NCBI Taxonomy database and the species taxon ID of the associated ENA records. The results are shown in Table 1, and show that despite the fact that the pipeline was focused on mining plant genomic regions, potential amplicons with minimal amounts of gaps/mismatches in the primer annealing regions (the maximum number was set at 2 per primer in the ecoPCR analysis) can be found in non-plant organisms for most of these primers. Some exceptions are as expected, such as the Light-harvesting chlorophyll protein, involved in photosynthesis, and a RGP family member, which are a set UDP-L-arabinose mutases involved in plant cell wall biogenesis. Other targets represent poorly characterised proteins, for which few instances have been sequenced and deposited in the sequence records.

### Output validation

**Fig 2:**
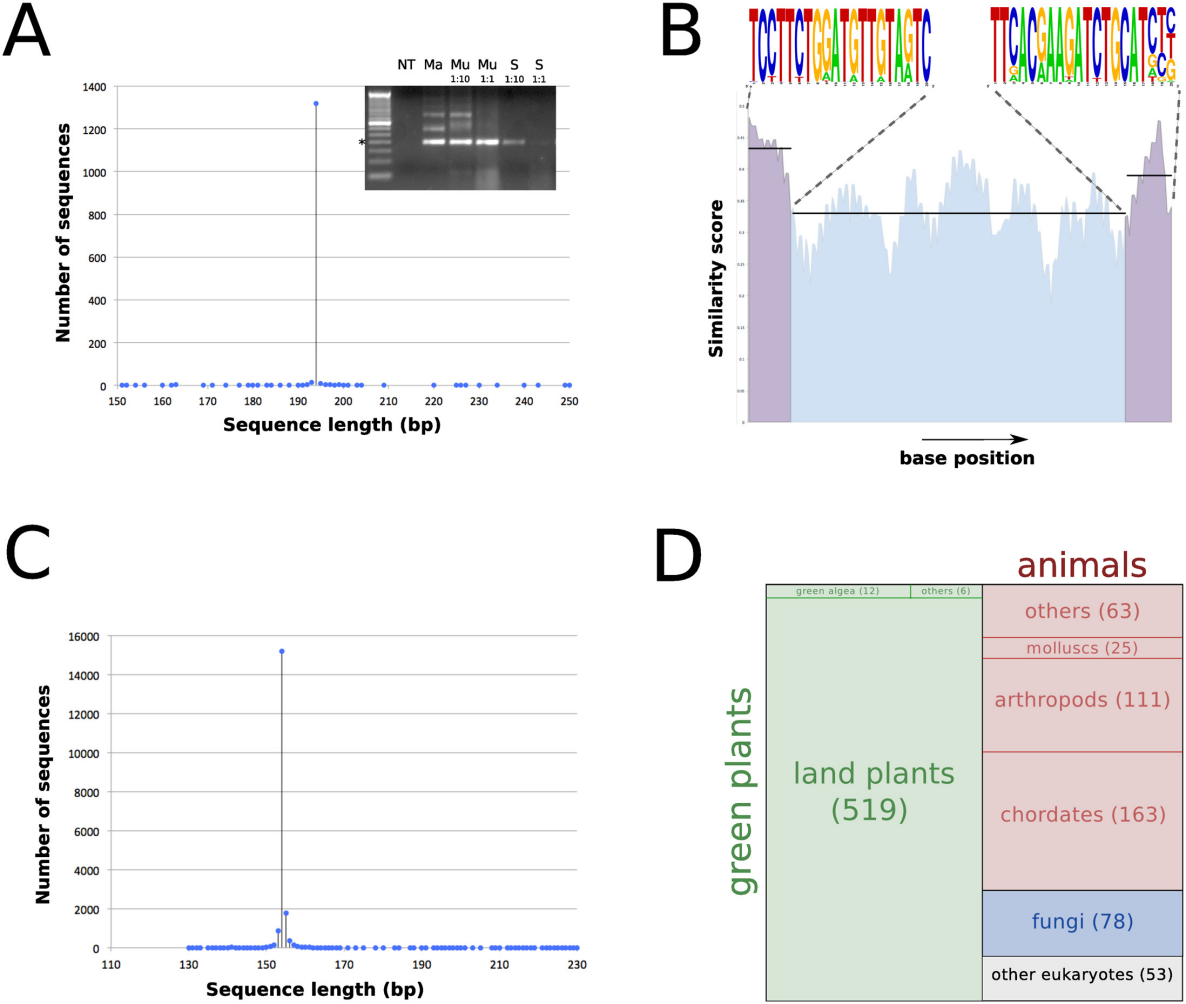
Analysis of the output produced by the pipeline using LIME sequences as an input. (A) Distribution of the amplicon lengths produced by *in silico* PCR on plant genomes. The inset shows a sample agarose gel obtained with the primers on various samples (NT: no template, Ma: Maize, Mu: Muesli, S: Strawberry), with the main band the expected size (the * shows the 200bp marker on the DNA ladder). (B) Consensus of all the amplicon sequences of size 194bp, showing the higher conservation in the primer binding sites (purple) compared to the intervening region (blue). The primer consensuses, produced by Weblogo, are also shown. (C) Distribution of the amplicon lengths produced by *in silico* PCR on the whole ENA nucleotide database. (D) Number of species for which at least one unique amplicon can be produced by the 23579-aaa primer pair, shown to relative scale.

The set of reference sequences, obtained by *in silico* PCR simulations with the primer set 23579-aaa on plant genomes (Fig 1G), are almost all of the identical length of 194bp (Fig 2A), suggesting a high specificity for the genomic regions amplified by these primers. Analysing the relative conservation at each position in these amplicon sequences, it is evident that, as expected, the primer binding sites are more conserved than the central region (Fig 2B). These primers target an exon in proteins of the Ubiquitin family, according to annotations of the different genomes in Ensembl. As described above, the primers were used to extract additional amplicons from the ENA database. This way, another 19000 additional reference sequences were extracted. Almost all of these sequences were of the expected size when taking into account the fact that ecoPCR removes the primer sequences from the amplicons (Fig 2C). These sequences, pooled with the ones extracted from the plant genomes (from which the primer regions were also removed), constitute the final set of reference amplicons for this primer pair. The species with at least one unique sequence now reach more than 1000, the majority of which are green plants, but they also now include many sequences from animals and fungi (Fig 2D).

#### Pure samples

The 23579-aaa primer pair was used to test various samples, described in the Materials and Methods section (maize, soybean, rice and strawberry). The barcode regions were amplified from the purified DNA, and bands of the expected size were obtained (see inset of Fig 2A). Some optimisation was sometimes necessary, mostly in terms of initial template concentration due to some PCR inhibition observed at lower dilutions of the genomic DNA, as shown with the increase in signal intensity at 1:10 template dilution compared to using undiluted template (Fig 2A). Once optimised, the PCR reactions products were sequenced by Next Generation Sequencing (NGS) using a Roche GS-Junior instrument. The reads were trimmed to remove the primer regions and combined with the >19000 referenced reads. This final set of sequences was clustered to pool in the same clusters sequences that are 100% identical, both in sequence and size. Clusters containing NGS reads and at least one reference read were the main focus of the subsequence analyses, from which the number of NGS reads and the species of the reference reads were recorded. An example analysis, using the maize (*Zea mays*) sample, is shown in Table 2.

**Table 2:** Barcode analysis of a Zea mays sample following clustering of the NGS reads with the reference sequences at 100% identity. The 21 clusters including at least one NGS read and at least one reference sequence are shown, ordered by the number of NGS reads. When reference reads from more than one species were found in the same cluster, the conclusion is shown as the highest taxonomy common to all the species.

**Cluster**	**# NGS reads**	**# Reference reads**	**Reference read(s) species**	**Conclusion**
1	6602	2	*Zea mays* (2)	*Zea mays*
2	3652	1	*Zea mays*	*Zea mays*
3	2806	1	*Zea mays*	*Zea mays*
4	515	1	*Zea mays*	*Zea mays*
5	348	1	*Zea mays*	*Zea mays*
6	79	1	*Oryza brachyantha*	*Oryza brachyanta*
7	24	1	*Zea mays*	*Zea mays*
8	21	2	*Triticum aestivum*, *Zea mays*	Poaceae
9	19	1	*Zea mays*	*Zea mays*
10	10	1	*Zea mays*	*Zea mays*
11	3	1	*Zea mays*	*Zea mays*
12	3	1	*Zea mays*	*Zea mays*
13	3	1	*Zea mays*	*Zea mays*
14	2	1	*Zea mays*	*Zea mays*
15	2	2	*Triticum aestivum*, *Zea mays*	Poaceae
16	2	1	*Zea mays*	*Zea mays*
17	2	1	*Zea mays*	*Zea mays*
18	1	1	*Zea mays*	*Zea mays*
19	1	3	*Panicum virgatum*, *Triticum aestivum*, *Zea mays*	Poaceae
20	1	1	*Zea mays*	*Zea mays*
21	1	1	*Zea mays*	*Zea mays*

**Fig 3:**
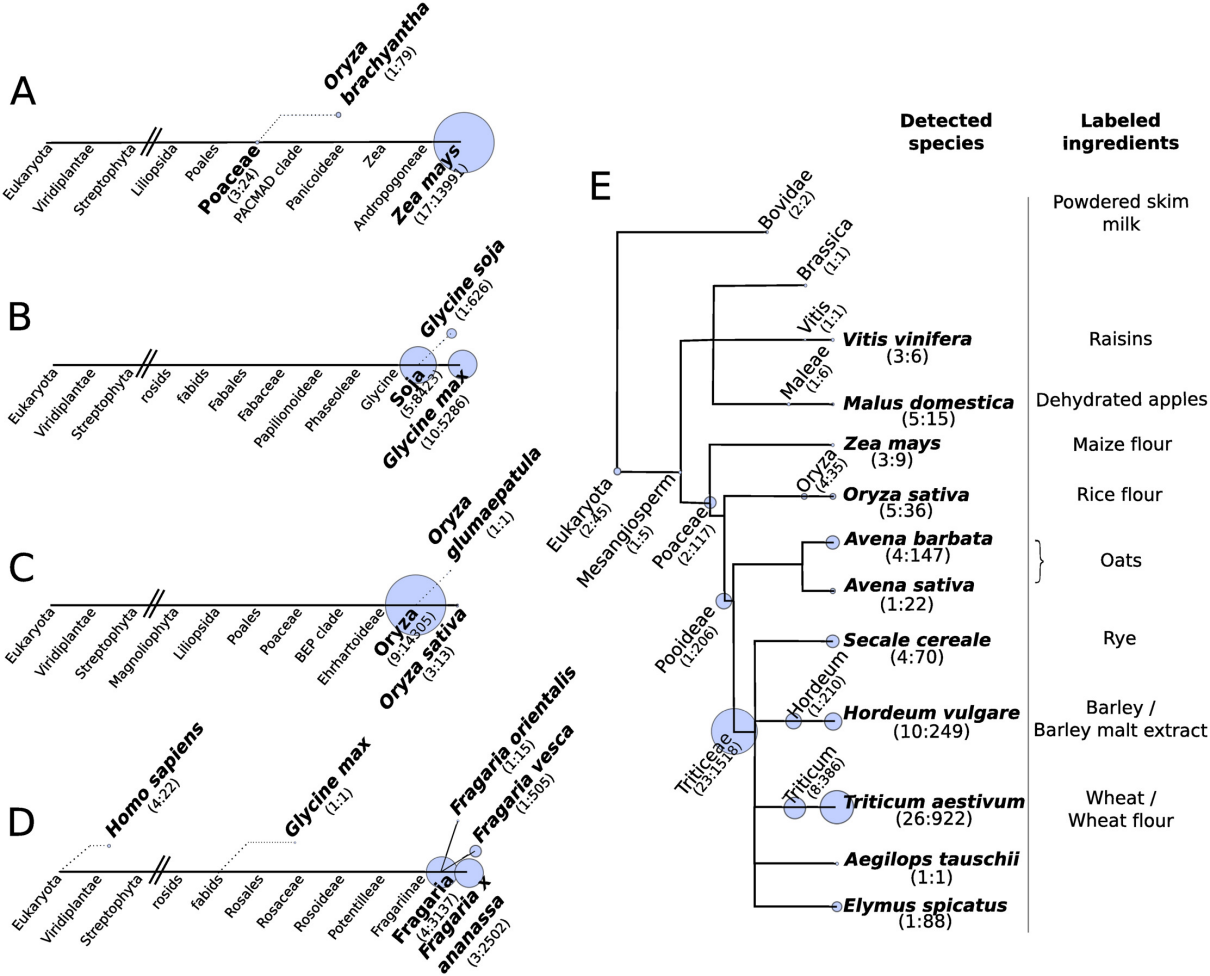
Species detection in various samples using the 23579-aaa barcoding primers. The tested samples were (A) Purchased maize (*Zea mays*) DNA. (B) Purchased soya (*Glycine max*) DNA. (C) A leaf from a rice (*Oryza sativa*) plant. (D) Fresh strawberries from the supermarket (*Fragaria x ananassa*). (E) A commercial pack of fruit and cereal muesli. The results are shown on a simplified taxonomy line, with the number of NGS reads assigned to that specific location as a blue circle whose area is proportional to the numerical value. The values in parenthesis represent the (number of clusters:total number of reads).

This analysis shows that 21 clusters were found to contain both at least one reference read and at least one NGS read. The 5 largest clusters, corresponding to the majority of the reads, all contain reference reads from *Zea mays*. In a few clusters, two or more reference reads were found (clusters 8, 15 and 19), as these sequences are reported to be identical in more than one species. For these, it was decided to assign, as a conclusion, the highest taxonomy common to all the species, as determined using NCBI’s Common Tree tool [41]. Only one cluster did not contain a *Zea mays* reference read, cluster 6, where 79 NGS reads were found to be identical to a reference region from *Oryza brachyantha*. This could be caused either by a trace contamination of the original sample or by a specific polymorphism in this region not captured in the currently available sequence information. The analyses in Table 2 are shown graphically in Fig 3A, aligned on a simplified taxonomy of *Zea mays*, with an indication of the relative amount of reads that could be assigned to each location.

Not all the reads produced could be assigned to a reference read, i.e. clusters were found containing NGS reads but no reference. In this case, the unassigned reads comprised about 28% of the total reads produced (5660/19757), a ratio representative of the average in all our experiments. Analyses of these clusters show that these reads fall in three different categories. First, as expected, many small clusters contained reads with 1 or 2 differences compared to one of the reference reads. Because the clustering is set at 100% identity, reads containing sequencing errors not corrected by the coral script during processing will end up being unassigned. Second, products of non-specific PCR amplification (see gel in Fig 2A) will also be sequenced, but not mapped to any reference. Third, some of the reference sequences, extracted bioinformatically from the genome sequences and the ENA nucleotide database, could be incorrect. The biggest unmapped cluster in our experiment, composed of 473 identical reads but no reference, has as a closest match a *Zea mays* reference read in our database to which no NGS reads were mapped (Chromosome 5:82481545-82481698), with two nucleotide differences. Most probably, this represents errors or polymorphisms in the genome sequence in this region.

The same experiment and analyses were performed on three additional pure samples. In a soybean (*Glycine max*) DNA sample (Fig 3B), the large majority of the reads mapped to *Glycine max* or its Soja subgenus, with a minority of reads assigned to the wild relative *Glycine soja*. A rice (*Oryza sativa*) sample (Fig 3C) displayed the almost totality of the reads mapped to the Oryza genus. Still, some of the reads (13 in total, in three independent clusters) could be mapped to a reference specific for *Oryza sativa*. Finally, testing strawberries bought from the supermarket (Fig 3D) resulted in the majority of reads being identical to reference reads from *Fragaria x ananassa* or to the Fragaria genus. Interestingly, this sample also showed 4 sets of NGS reads identical to independent *Homo sapiens* references, for a total of 22 reads, most probably illustrating the fact that the fruits were manipulated by hand prior to DNA extraction and testing.

#### Relative quantification

We then tested the possibility of achieving a relative quantification of different species in the same sample, by testing mixtures of DNA from maize and soybean at 25:75, 50:50 and 75:25 weight ratios. Simply using, for this goal, the total number of reads assigned to each species was felt to be inadequate, for various reasons. First, the number of copies of the target regions can vary from species to species. In this case, the bioinformatics analyses of the genomes showed 19 potential amplification sites for maize (with the limit set at 4 total gaps and mismatches in the primer annealing sites), compared to 13 for soybean. In addition, not all reference sequences are specific at the species level, with some being identical between different species. Finally, Table 2 showed a large variation in the number of NGS reads identical to the individual instances of the barcode region within the same genome, for reasons that most probably relate to varying efficiency of the PCR amplification with the barcode primers. For these reasons, we selected, for each species, the reference that showed the largest cluster, with a single reference read in the individual samples tested above. The assumption, in this case, was that this specific genomic locus would have the highest probability of being closest to optimum PCR amplification efficiency, hence comparable between species. This corresponded to EMBL record AI677436 for *Zea mays* (Cluster 2 in Table 2) and Chromosome 1:3082442-3082595 for *Glycine max* (data not shown). When comparing the number of NGS reads clustering to these references in the mixed DNA samples, the relative ratios, once corrected for the genome sizes, are very close to the expected ones (Table 3).

**Table 3:** Relative quantification in mixed maize:soy samples. Purified DNA from the two species were mixed in the shown weight:weight ratio prior to barcode PCR and sequencing. The number of reads reported corresponds to the reference read selected based on the results of the individual samples (see text for details), corrected for the C values of both species.

	NGS reads		Corrected NGS reads		
**Original sample (maize:soy)**	**soy**	**maize**	**soy**	**maize**	**Experimental maize:soy**
25:75	2688	442	3037,44	1206,66	28,4:71,6
50:50	1396	667	1577,48	1848,21	54,0:46,0
75:25	831	1103	939,03	2765,49	74,7:25,3

#### Complex sample

Finally, we tested the primer pair in a complex sample, a bag of muesli bought from the supermarket. A sample, containing a mix of cereals and fruits, was homogenised, the DNA extracted and the barcoding experiment was performed as for the pure samples described above. A summary of the results obtained is shown in Fig 3E. A large number of clusters were obtained, almost exclusively with reference reads of plant origin and mapping to the species corresponding to the labelled ingredients (or within their taxonomy). The only reads assigned outside of the plant kingdom are two clusters mapped to Bovidae, possibly the result of the detection of the powdered milk. The main species detected but not found in the ingredient list is *Elymus spicatus* (a species of grass), to which 88 reads were assigned. Whether this is a legitimate contamination or reflects a gap in the references is unclear. The only ingredients of eukaryotic origin listed on the packaging but not detected in our experiment were concentrated lemon juice, non-hydrogenated vegetable oil (sunflower) and soy lecithin, although the processing level for each of these might impede detection with DNA-based methods.

## Discussion

The work presented here demonstrates an integrated strategy for the identification of novel nuclear genomic barcode primer sets that takes advantage of recent advances in bioinformatics technologies and availability of plant genome information, together with the experimental demonstration of the efficacy of this strategy. Applied systematically, this pipeline could lead to the development of panels of barcode primers tailored for specific purposes that could complement and improve the currently used mitochondrial or plastid targets. The main aim of the pipeline is to identify new candidate regions to be used for barcoding experiments, and design primer pairs within those regions. The use of these primers for species identification experiments requires a set of reference sequences to which the sequenced fragments can be compared, and the last part of the pipeline (Fig 1G and the ecoPCR scan of the ENA database) aimed to build such a set for the purpose of testing the output. This worked reasonably well in our experiments (Fig 3). However, as for every barcoding framework, it remains important, once new primers are chosen, to establish the correctness of, and expand, the reference sequences for the set of species of interest (as is done, for example, in the Barcode of Life project [10])by testing samples of certified taxonomic origin.

An example of the added value of nuclear barcode targets was that it allowed in a single experiment the identification of both mammalian and plant species (human manipulation of the strawberry sample, and milk in the muesli, Fig 3D and 3E). This was predicted from extending the bioinformatics extraction of potential reference sequences to the whole records of the ENA std database, using the ecoPCR tool that was designed for this purpose (Fig 2D and Table 1). Testing the other primers pairs showed a varying amount of spread across the plants/invertebrates/vertebrates divisions. Unlike the PCR simulations on whole genomes, the identification of records in the ENA database depends on the submission of specific sequence records to the database, explaining the small number of records for uncharacterised or poorly studied proteins. Others, like the Reversibly Glycosylated Proteins (RGP) family member and the Light-harvesting chlorophyll protein complex subunit were expected to be specific for plant species, and the distribution in the ENA database reflects this. More unexpected is the primer pair targeting an HSP70 family member, who, despite the ubiquituous role and presence of HSP proteins in eukaryotes, seems also limited to plant species, the only exceptions being *Aedes aegypti* (mosquito, accession DW993187), *Rhynchophorus ferrugineus* (red palm weevil, accession JR467708) and *Cryptosporidium muris* (a rodent pathogen, accession FD387861). This can either be due to the fact that the actual HSP70 family member targeted by this primer pair is one that evolved specifically in plants, or that the primer pairs were designed in a region more conserved in plants compared to other organisms, where the number of mismatches and gaps could be above the thresholds set in the pipeline. In both these cases, this would be due to the exclusive use, in the different steps shown in Fig 1, of plant species genomes. If the aim was to increase the broad specificity of the primers produced by the pipeline, the use of more varied genomes could be considered.

As a second added value, and since the copy number of genomic references between species is much easier to control than mitochondria or plastids, these primers show promising results for relative quantification of mixed ingredients, assuming a preliminary assessment of the corresponding pure samples in order to select a specific reference read (Table 3).

The method we have chosen to assign a sequenced read to a species was through clustering at 100% identity across the whole reference sequences. This will obviously cause some reads to be unassigned, either due to sequence polymorphisms in the barcode region or errors in the sequencing. Indeed, despite correction of the sequencer output with specialised scripts such as coral (see Materials and Methods), all our analyses showed a pool of unassigned reads (data not shown). Although the main goal of this article is to present the strategy designed to produce novel barcode candidates, it is obvious that the integration of these novel barcodes in a correct, sensitive and accurate framework for species identification will require strategies that take into account all these factors; see for example, Bruno et al, 2015 [42].

In our experiments, the ratio of reads assigned to the species compared to the genus level varied depending on the number of close relatives sequences found in the public sequences databases, in particular for the extreme cases of *Zea mays* (most reads assigned to the species level) and *Oryza sativa* (most reads assigned to the genus level). The high amount of duplication that occurs in the nuclear genomes, often described as an impediment of finding nuclear DNA barcode candidates, becomes an advantage here, as increasing the copy number of the barcode DNA region in each species increases the chances of finding a location that has diverged between close relatives.

The first step of the pipeline, seed extension (Fig 1A), is also a screening step for the input seed sequences, as sequences that do not produce a hit in either of the two genomes are dropped. We chose at this step to use the genomes of one dicot, *Arabidopsis thaliana*, and one monocot, *Oryza sativa*, to avoid a bias for one or the other clade. This seems to have been the case, at least when evaluated following sequence extraction (step described in Fig 1D, see S1 Text). We have tested experimentally, as pure samples, two monocots (*Zea mays, Oryza sativa*) and two dicots (*Glycine max, Fragaria x ananassa*), all with good results. Testing the mixture of *Zea mays* and *Glycine max* also showed no bias towards one or the other. Only in the complex sample (Muesli) did we notice an imbalance between the monocots (the cereals) and the dicots (the fruits), but this is most probably due to a combination of the ingredient ratio (the package lists less than 10% fruit content) and of a lower DNA extraction efficiency of the dried fruit compared to the cereal component of the mix.

It should also be emphasized that no specific objectives were taken into consideration when choosing the primer set to be tested experimentally. Future work in this project, in addition to the generation of additional barcode candidates with more input seeds and improved sequence information, will involve the compilation of the information for each identified primer pair and their associated reference sequences in publicly available databases that can be queried for specific aims, for example the identification of barcode primers with a high resolution power between specific species or within a specific genus. This strategy would then allow the development of tailored barcoding solutions, involving different targets and primer sets, able to address specific gaps in species identification in complex samples and support the proper implementation of regulations aiming at guaranteeing the safety and protection of consumers.

## Supporting Information

S1 Text Supplementary information: Detailed bioinformatics pipeline. This supplementary information includes a detailed description of the bioinformatics pipeline summarized in the text and in Fig 1, as well as the intermediate results obtained with the LIME sequences used as input.
(PDF)

S1 Scripts Supplementary information: Scripts of the bioinformatics pipeline. This supplementary information includes a set of scripts, written in the Ruby language, that was used to generate the data presented in this article.
(ZIP)
