# Supplementary material for: Towards Plant Species Identification in Complex Samples: A Bioinformatics Pipeline for the Identification of Novel Nuclear Barcode Candidates
Source: PLoS One. 2016 Jan 25;11(1):e0147692. doi: 10.1371/journal.pone.0147692 (PMC4725681; doi:10.1371/journal.pone.0147692)

Supplementary information for ”Towards plant species identification in complex samples: a bioinformatics pipeline for the identification of novel nuclear barcode candidates.”

Angers-Loustau et al.

The main plants genomes used for the analyses were downloaded from Ensembl, and included all the available genomic sequences of release 23 (from Ensembl's FTP server at ftp://ftp.ensemblgenomes.org/pub/plants/release-23). In addition, available genomes not found in Ensembl were downloaded from the Genbank ftp genome repository (ftp://ftp.ncbi.nih.gov/genomes/genbank/plant/, accessed 14-10-2014). When more than one version, or subspecies, were available for the same species, the largest file was kept. The full list of genomes is listed in Table A. Scripts were generated in Ruby to make use of publicly available command line binary tools, installed locally (see Methods section of the main article for details) The seeds to be sent in the pipelines were obtained from a published dataset of 1039 plants complex Long Identical Multispecies Elements (LIMEs) characterised and published by Reneker et al. (2012)[1], transformed into a multiFASTA file in which only one copy of each LIME was kept. The numbering scheme of each sequence was maintained from Reneker et al. The final file contained 1039 unique sequences.

1 Seed extension

Seed sequences for the pipeline can include any sequence thought to be identical or very similar between different species. The first step in the pipeline is then the extension of each of the seed sequences to include neighbouring regions where,

though not necessarily identical, the sequence would be similar enough to be used as DNA barcodes. To allow a script to perform this step automatically for the seed sequences, a two-genome strategy was designed, using *Arabidopsis thaliana* (a dicot) and *Oryza sativa* (a monocot). These two genomes were selected as they are both relatively short (i.e non-repetitive) and phylogenetically quite distant from each other.

One requirement for this step is then that the seed sequence is present in both of these genomes, which was not the case for all LIMEs; however, the great majority (795/1039, more than 75%) could be extended with this strategy.

The idea behind this extension is summarized in Figure A and includes, for each seed sequence:

1. A BLAST search is performed using the seed sequence as a query against the *A. thaliana* genome (protocol: blastn, no soft masking, e-value set at 1E-05)

2. The coordinates of the top hit are parsed, and extended to include the sequence before and after the identified genomic region. The corresponding sequence is extracted from the genome using these new coordinates

3. A second BLAST is performed using this extended sequence as a query against the *O. sativa* genome, with the same parameters as step 1.

4. The coordinates of the top hit are parsed, and the corresponding sequence extracted. The sequence of the query is also trimmed to keep the corresponding interval.

The result of this two-step BLAST analysis is the extension of the seed sequences to include neighbouring sequences that, although not identical, are significantly similar in at least the two genomes tested.

For the LIMEs, an extension of 1kb (on each side) was originally chosen, resulting in an increase in the average sequence length from 150bp to 1.4kb, as seen in Figure B. However, this plot also shows that the extension was limited by the value chosen, with a concentration of sequences along the ”Original Length + 2000bp” line (dashed line).

The step was thus repeated with an extension of 2kb (on each side). Figure B shows that, with this value, there is a greater spread of the resulting sequences, with no indication of limitation at the upper sizes. The average sequence length reached 1.988kb.

The resulting pairs of sequences are saved in individual multi-FASTA files, one for each original seed, and used for the subsequent steps of the pipeline.

2 Removal of duplicate seeds

Figure B shows that, once the LIMEs sequences have been extended, the resulting sizes show a tendency to stratify at specific sizes (see, for example, a 3000bp on Figure B). These generally represent duplicate sequences, which happens even when unique seeds are fed in the pipeline, if some of them are close enough on the genomes to be merged by the extension step.

To eliminate the duplicates, the sequences are all compared to each other by BLAST and pooled in groups with significant similarity scores (i.e. e value $<$ 1E-50). The results are shown in Table B, and show that only 86 extended sequences from the original 795 LIMEs remain unique, with the others clustering in groups of two up to 177 sequences.

Only one sequence from each group was kept for future characterisation, for a total of 150 sequences. In the great majority of the cases, the members within the same group were all the same size, suggesting that the sequences were identical. When this was not the case, the longest sequence of the group was kept.

3 Genomes screen

Each unique pair of extended sequences was used to screen each of the 140 plants genomes shown in Table A to identify sequences with high similarity.

In brief, the two sequences were aligned using Clustal Omega, and the output (in the Stockholm format) saved in an intermediate file. This file was then used to build a Hidden Markov Model file (using the hmmbuild tool from the HMMER suite), allowing, using the nhhmer tool, to scan each of the plant genomes, producing 21000 output files (150 sequences x 140 genomes).

This computer-intensive step was performed on a cluster made of 32 computing nodes (HP Blates BL460 G6 with Infiniband QDR interconnect), each equipped with 2 Quad-Core Intel Zeon E5540 (2.53GHz) processors and 24GB of RAM. The requests were managed with a Condor workload scheduler, with a separate process launched for each combination of hmmer file and genome, for a total of 233472 cpu-hours in 38 days. The nhmmer scripts were launched with the following parameters: –noali (no alignments in the output file), –max (Turn

all heuristic filters off (less speed, more power)). The results files were pooled in a single folder for further analyses.

The content of these output files was evaluated in order to determine the criteria for hits selection. The two main criteria analysed were the length of the hit found (as expressed as a percentage of the length of the extended sequences) and the accuracy score produced by nhmmer, both parsed from the output files using custom ruby scripts. Figure C shows the number of hits that are above a specific accuracy value (0.5 to 1.0, in 0.1 increments). The different lines correspond to different length threshold values (65% up to 100%, in 5% increments). The curves show a linear increase in the number of sequences up to an accuracy threshold of about 0.95, below which the number of sequences grows at an increased rate. Using the double derivative of this curve shows that the inflexion occurs at an accuracy of 0.94, which was then kept for future steps.

To determine the length threshold, a similar plot was produced (Figure C). The graph shows a large increase of sequences when lowering the threshold from 100% to 92% of the original extended sequences (dashed line), before increasing at a linear rate. For this reason, at least 92% of the input sequences length was chosen as the threshold for future steps.

4 Sequence extraction

Using the thresholds established from the analysis of the nhmmer output files, the files were once again parsed in order to extract the coordinates (i.e. chromosome, start, end and strand details) of the matching hits from all the genomes (Ensembl and NCBI), which were then fed into the extractseq tool of the EMBOSS suite to extract the sequences from the original genome FASTA files.

The output of this step was a set of 150 multi-FASTA files, one for each unique extended LIME, and containing all the regions of high sequence similarity found in the plants genomes, with an assigned name that links each of the sequences to the genome and genomic location of origin.

The first analysis done on the Conserved Sequences was to evaluate the number of sequences extracted from the genomes of each of the analysed species. The results are shown on Figure D, with each of the sequences in the rows and the species in the columns, ordered first based on the number of species with at least one sequence, then by the average number of sequences per species. Figure D shows that the numbers go from one extreme, i.e. only one sequence from *Oriza sativa* and one sequence from *Arabidopsis thaliana*, which was the minimal se-

lection criteria in step 2, to the other, i.e. hundreds of sequences from most of the species. Possibly due to the extension step in *Oryza sativa* and *Arabidopsis thaliana* in step 2, none of the sequences were found to be specific for or enriched in monocots or dicots. However, relatively few sequences were extracted from the "other" genomes, in particular from algea. Some genomes, such as *Amaranthus tuberculatus* and all the six *Persemon* family members are also greatly under-represented, possibly reflecting the yet incomplete nature of their genome sequences.

5 Sequence alignment

For each set of sequences, an alignment was produced using MAFFT[2], and the resulting file processed using the Weblogo script[3], which is normally used to generate graphical representations of amino acid or nucleic acid multiple sequence alignments, but can also produce a text output showing the DNA base count and non-gaps frequency at every position of a set of aligned sequences. This table ouput was used for the next steps of the pipeline.

6 Identification of candidate primers

For each of the set of conserved sequences, the Weblogo output file is processed to determine the optimal regions to design primer sequences. A script scans the positions and identifies regions of 20 consecutive bases identical in at least 80% of the sequences of the alignment. Every time such a region is identified, the same analysis is made starting at position +300, scanning back until position +150 for another 20 base region with the same characteristics. If a reverse primer is found, the primer pair sequences are extracted and saved. This step thus generates sets of primers, for each of the conserved sequences, that would produce amplicons between 150 and 300 base pairs

With the LIMEs input, this step produced 18,915 primer pairs to be analysed in the subsequent steps.

7 Amplicon scan in plant genomes

The potential amplicons produced by the candidate primer pairs were determined through in silico simulation of PCR on the 140 plant genomes from Table A. The

genomes were used instead of the sequences extracted after step 4 in order to both compensate for any sequence not identified during the HMM scan and to correct for the fact that the amplicon region is shorter than the original expanded seed, and thus additional similar regions could be present in the genomes.

The primer were formatted for analyses using the e-PCR tool from NCBI[4] by exporting them all into the same .sts file. This STS file was then analysed against each genome with the following parameters: -n= 2 (max mismatches allowed), -g=2 (max indels allowed), -f=3 (discontiguos words) -t=3 (tabular output format). The output file for each genome was then parsed and the hits sequences extracted using the extractseq tool.

The amplicon sequences produced by each primer pair were pooled in a multiFASTA file, and the name of each sequence incorporates the label of the primer pair that produced it, the genome file (including species name) from which it was extracted as well as the exact genomic location. It is possible, at this stage, to have identical amplicon sequences, either within the same species or between different species.

8 Barcodes analysis

This step involves the final selection of primers, from the candidates produced by the pipeline, through the analysis of the amplicon sequences produced in the previous step. The details and criteria depend on the expected purpose of the barcode primers, and can vary.

For the current study, in order to test the output of the pipeline generated from the LIMEs seeds, we have chosen to order the primer pairs by the number of species for which at least one unique barcode sequence was found. Other strategies can include evaluating the intra- and inter-species variability within a genus or between different species that need to be specifically identified for a specific purpose.

For each barcode primer, to find the number of species for which it produce at least one unique barcode, the multiFASTA file was clustered using cd-hit-est with the following option: -n 8 (word length), -c 1.0 (sequence identity) -g 1 (clustering mode) -aL 1 (alignment coverage for the longest sequence) -aS 1 (alignment coverage for the shortest sequence). The resulting cluster file was parsed to identify the clusters containing a single species, whose identity was then pooled.

The reference amplicon list was expanded with the sequence from the whole ENA database, which was downloaded locally (release 123, downloaded 7/03/2015)

and formatted for ecoPCR analyses with the supplied ecoPCRFormat.py script. The resulting database was then scanned with the ecoPCR script, with the options -e2 (max errors allowed by oligonucleotide) and length limits plus or minus 50bp from the average amplicon length extracted from the genomes. The output file, that already includes the extracted amplicon sequences, was parsed to generate multiFASTA files, and each amplicon sequence was assigned a name including the species and original accession number. The resulting numbers are shown in Figure 2A and Table 1 of the main article. Table C shows the primer sequences of the primers in Table 1 of the main article.

References

1. Reneker, J. *et al.* Long identical multispecies elements in plant and animal genomes. *Proceedings of the National Academy of Sciences* **109**, E1183–E1191 (2012).

2. Katoh, K., Misawa, K., Kuma, K.-i. & Miyata, T. MAFFT: a novel method for rapid multiple sequence alignment based on fast Fourier transform. *Nucleic acids research* **30**, 3059–3066 (2002).

3. Crooks, G. E., Hon, G., Chandonia, J.-M. & Brenner, S. E. WebLogo: a sequence logo generator. *Genome research* **14**, 1188–1190 (2004).

4. Schuler, G. D. Sequence mapping by electronic PCR. *Genome research* **7**, 541–550 (1997).

Supplementary Tables

Table A: List of plant genomes used in the bioinformatics pipelines, together with their origin and version

Scientific name	Origin	Version	Common name
Actinidia chinensis	Genbank	v1	Kiwi
Aegilops tauschii	Ensembl	23	Tausch's goat-grass
Aethionema arabicum	Genbank	VEGI_AA_v_1.0	
Amaranthus hypochondriacus	Genbank	AHP_1.0	Prince-of-Wales feather
Amaranthus tuberculatus	Genbank	ASM18065v1	Tall waterhemp
Amborella trichopoda	Ensembl	23	Amborella
Amborella trichopoda	Genbank	AMTR1.0	
Aquilaria agallochum	Genbank	v1	
Arabidopsis halleri	Genbank	subsp._gemmifera_Ahal_1.0	
Arabidopsis lyrata	Ensembl	23	Lyrate rockcress
Arabidopsis thaliana	Ensembl	23	Thale-cress
Arabis alpina	Genbank	V4	Alpine rock-cress
Auxenochlorella protothecoides	Genbank	ASM73321v1	A green algae
Azadirachta indica	Genbank	AzaInd2.0	Neem tree
Beta vulgaris	Genbank	RefBeet-1.2.1	Beet
Betula nana	Genbank	ASM32700v1	Dwarf birch
Brachypodium distachyon	Ensembl	23	False brome
Brassica napus	Genbank	v1	Rapeseed
Brassica oleracea	Ensembl	23	
Brassica rapa	Ensembl	23	Field mustard
Cajanus cajan	Genbank	Asha_ver1.0	Pigeon pea
Camelina sativa	Genbank	Cs_genomic	Camelina
Cannabis sativa	Genbank	canSat3	
Capsella rubella	Genbank	Caprub1_0	Pink shepherd's purse
Capsicum annuum	Genbank	PGAv.1.5	Bell/chilli peppers
Carica papaya	Genbank	Papaya1.0	Papaya
Castanea mollissima	Genbank	ASM76360v1	Chinese chestnut

Table A – continued from previous page

Scientific name	Origin	Version	Common name
Chlamydomonas reinhardtii	Ensembl	23	A green algae
Chlorella variabilis	Genbank	v_1.0	A green algae
Cicer arietinum	Genbank	ASM33114v1	Chick pea
Citrullus lanatus	Genbank	CiLa_1.0	Watermelon
Citrus clementina	Genbank	v1.0	Clementine
Citrus sinensis	Genbank	Csi_valencia_1.0	Orange
Cleome hassleriana	Genbank	ASM46358v1	Spider flower
Coccomyxa subellipsoidea	Genbank	C-169_v2.0	A microalgae
Cucumis melo	Genbank	ASM31304v1	Muskmelon
Cucumis sativus	Genbank	CSB10A_v1	Cucumber
Cyanidioschyzon merolae	Ensembl	23	A red algae
Dianthus caryophyllus	Genbank	DCA_r1.0	Carnation
Elaeis guineensi	Genbank	EG5	African oil palm
Elaeis oleifera	Genbank	EO8	American oil palm
Ensete ventricosum	Genbank	v1.1	Ethiopian banana
Erycina pusilla	Genbank	EpS81.0	An orchid
Eucalyptus camaldulensis	Genbank	EUC_r1.0	River Red gum
Eucalyptus grandis	Genbank	Egrandis1_0	Flooded gum
Eutrema parvulum	Genbank	v01	
Eutrema salsugineum	Genbank	Eutsalg1_0	
Fragaria iinumae	Genbank	FII_r1.1	A strawberry
Fragaria nipponica	Genbank	FNI_r1.1	A strawberry
Fragaria nubicola	Genbank	FNU_r1.1	A strawberry
Fragaria orientalis	Genbank	FOR_r1.1	A strawberry
Fragaria vesca	Genbank	FraVesHawaii_1.0	Wild strawberry
Fragaria x ananassa	Genbank	FAN_r1.1	Garden strawberry
Fraxinus excelsior	Genbank	BATG-0.4	European ash
Galdieria sulphuraria	Genbank	ASM34128v1	A red algae
Genlisea aurea	Genbank	1.0	
Glycine max	Ensembl	23	Soybean
Glycine soja	Genbank	W05v1.0	Wild soybean
Gossypium arboreum	Genbank	v1.0	Tree cotton
Gossypium raimondii	Genbank	Gr_v1.0	
Helicosporidium sp.ATCC 50920	Genbank	v1.0	A green algae

Table A – continued from previous page

Scientific name	Origin	Version	Common name
Hevea brasiliensis	Genbank	1.0	Par rubber tree
Hordeum pubiflorum	Genbank	assembly1	
Hordeum vulgare	Ensembl	23	Barley
Jatropha curcas	Genbank	JatCur_1.0	Barbados Nut
Klebsormidium flaccidum	Genbank	ASM70883v1	A green algae
Lactuca sativa	Genbank	Legassy_V2	Lettuce
Lagenaria siceraria	Genbank	Bottle_gourd_genomic	Bottle gourd
Leavenworthia alabamica	Genbank	VEGI_LA_v_1.0	Alabama glade-cress
Leersia perrieri	Ensembl	23	Leersia
Linum usitatissimum	Genbank	LinUsi_v1.1	Flax
Lotus japonicus	Genbank	ASM18111v1	
Lupinus angustifolius	Genbank	genome_scaffold	Blue lupin
Malus x domestica	Genbank	MalDomGD1.0	Apple
Manihot esculenta	Genbank	MK_v2b	Cassava (Manioc)
Medicago truncatula	Ensembl	23	Barrel medic
Micromonas pusilla	Genbank	CCMP1545_v2.0	An algae
Mimulus guttatus	Genbank	Mimgu1_0	Common monkey-flower
Morus notabilis	Genbank	ASM41409v2	Mulberry
Musa acuminata	Ensembl	23	Banana
Nelumbo nucifera	Genbank	1.1	Chinese Lotus
Nicotiana benthamiana	Genbank	Ni_ben	
Nicotiana otophora	Genbank	v1	
Nicotiana sylvestris	Genbank	Nsyl	Woodland tobacco
Nicotiana tabacum	Genbank	BX_v1	Tobacco
Nicotiana tomentosiformis	Genbank	Ntom_v01	
Oryza barthii	Ensembl	23	African wild rice
Oryza brachyantha	Ensembl	23	
Oryza glaberrima	Ensembl	23	African rice
Oryza glumaepatula	Ensembl	23	
Oryza granulata	Genbank	OgranChr3sV1	
Oryza indica	Ensembl	23	Indica rice
Oryza meridionalis	Ensembl	23	

Table A – continued from previous page

Scientific name	Origin	Version	Common name
Oryza minuta	Genbank	ASM63269v1	
Oryza nivara	Ensembl	23	
Oryza punctata	Ensembl	23	
Oryza rufipogon	Ensembl	23	Common wild rice
Oryza sativa	Ensembl	23	Japonica rice
Ostreococcus lucimarinus	Ensembl	23	A green algae
Ostreococcus tauri	Genbank	version_050606	A green algae
Penstemon centranthifolius	Genbank	ASM73743v1	Scarlet buglar
Penstemon cyananthus	Genbank	ASM28100v1	Wasatch Penstemon
Penstemon davidsonii	Genbank	ASM28098v1	Davidson's Penstemon
Penstemon dissectus	Genbank	ASM28096v1	Georgia Beard-tongue
Penstemon fruticosus	Genbank	ASM28102v1	Bush penstemon
Penstemon grinnellii	Genbank	ASM73742v1	Grinnell's beard-tongue
Phaseolus vulgaris	Genbank	PhaVulg1_0	Common bean
Phoenix dactylifera	Genbank	DPV01	Date palm
Physcomitrella patens	Ensembl	23	A moss
Physcomitrella patens	Genbank	V1.1	A moss
Pinus taeda	Genbank	PtaedaFosmidLib.0.8	Loblolly pine
Populus euphratica	Genbank	PopEup_1.0	Euphrates Poplar
Populus trichocarpa	Ensembl	23	Western balsam poplar
Prunus mume	Genbank	P.mume_V1.0	Chinese plum
Prunus persica	Ensembl	23	Peach
Pyrus x bretschneider	Genbank	Pbr_v1.0	Yea pear
Raphanus sativus	Genbank	RSA_r1.0	Radish
Ricinus communis	Genbank	JCVI_RCG_1.1	Castor oil plant
Selaginella moellendorffii	Ensembl	23	A spike moss
Sesamum indicum	Genbank	v1.0	Sesame
Setaria italica	Ensembl	23	Foxtail millet
Sisymbrium irio	Genbank	VEGI_SI_v_1.0	London rocket
Solanum arcanum	Genbank	Soarc10	Wild tomato

Table A – continued from previous page

Scientific name	Origin	Version	Common name
Solanum habrochaites	Genbank	Sohab10	
Solanum lycopersicum	Ensembl	23	Tomato
Solanum pennellii	Genbank	Sopen10	
Solanum pimpinellifolium	Genbank	Sol_pimpi_v1.0	Currant tomato
Solanum tuberosum	Ensembl	23	Potato
Sorghum bicolor	Ensembl	23	Sorghum
Spinacia oleracea	Genbank	Viroflay-1.0.1	Spinach
Spirodela polyrhiza	Genbank	v01	Common duckweed
Theobroma cacao	Ensembl	23	Cocoa
Trifolium pratense	Genbank	Tp1.0	Red clover
Triticum urartu	Ensembl	23	An einkorn
Vigna angularis	Genbank	var._angularis	Adzuki bean
Vigna radiata	Genbank	ver6	Mung bean
Vitis vinifera	Ensembl	23	Wine grape
Volvox carteri	Genbank	f._nagariensis_v1.0	A green algae
Zea mays	Ensembl	23	Maize
Zizania latifolia	Genbank	v01	Manchurian wild rice

Table B: Duplicate analysis of the extended LIME sequences

size of group	number of groups	number of sequences
1	86	86
2	28	56
3	9	27
4	3	12
5	5	25
6	2	12
7	1	7
8	2	16
10	2	20
14	2	28
17	3	51
26	1	26
27	1	27
31	1	31
39	1	39
29	1	29
126	1	126
177	1	177
**Total**	**150**	**795**

Table C: Sequences of the primers shown in Table 1 of the main article

Primer pair	forward/reverse primers
23579-aaa	5' TCCTTCTGGATGTTGTAGTC 3'<br>5' AAGATGCAGATCTTCGTGAA 3'
24772-aaa	5' CTCTCCAGCTCCTTCATCTT 3'<br>5' GATGAGGAGCACAAGAAGAA 3'
586-aaq	5' CTGCCAGTAGTCATATGCTT 3'<br>5' CTACCATCGAAAGTTGATAG 3'
25268-aaa	5' TGGTACTTCTTCAGCCACAA 3'<br>5' CGATACTCATGCATGATCCA 3'
23965-aaa	5' CGATACTCGTGCATGATCCA 3'<br>5' GAGAAAGAGTGGTACTTCTT 3'
18638-aqc	5' TATGACTGAACGCCTCTAAG 3'<br>5' TAAGTCGTCTGCAAAGGATT 3'
632-aio	5' AGGGAAGTCGGCAAAATGGA 3'<br>5' CACTTTGACATTCAGAGCAC 3'
637-aek	5' GCATCAGGTCTCCAAGGTGA 3'<br>5' ATTAAACAGTCGGATTCCCC 3'
24430-aab	5' CAGATCTTCAGCGAGGGTGG 3'<br>5' CCCTTTCCGGTGACAATGGC 3'
24079-aae	5' TGATTGGTGGGGTGCGCCAG 3'<br>5' TCATACCTTTCTCGGCCTTT 3'
25227-aay	5' ATCCCTGACTATGATGCTCC 3'<br>5' CACCAACCAGCCCACATGTC 3'

Supplementary Figures

Figure A: Graphical representation of the strategy for the ”Seed extension” step of the pipeline

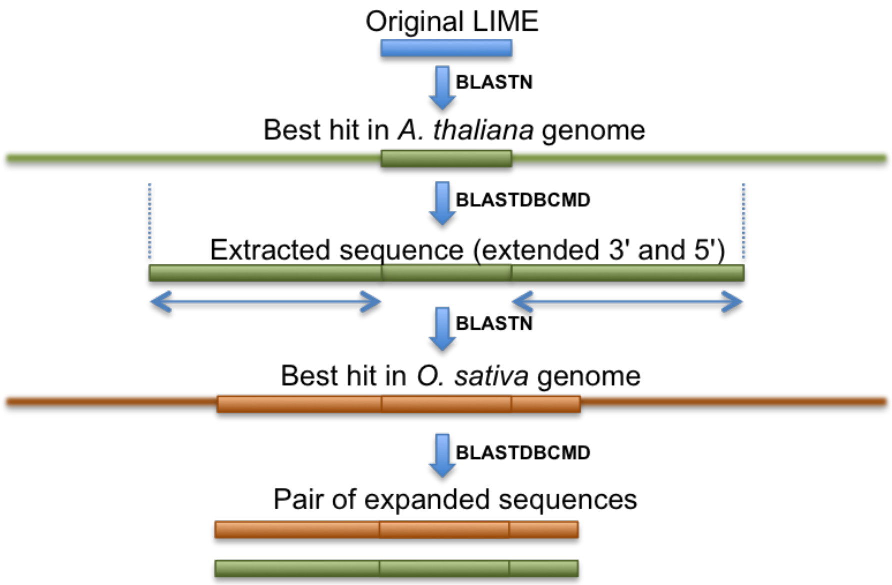

Figure B: Plot of the sequences lengths before (horizontal axis) and after (vertical axis) the extension step. A) Extending by 1kb on each side shows a concentration of sequences along the maximum theoretical length line (grey dash). B) Extending by 2kb on each side shows an improved spread of sequence length, with no indication of a limiting factor.

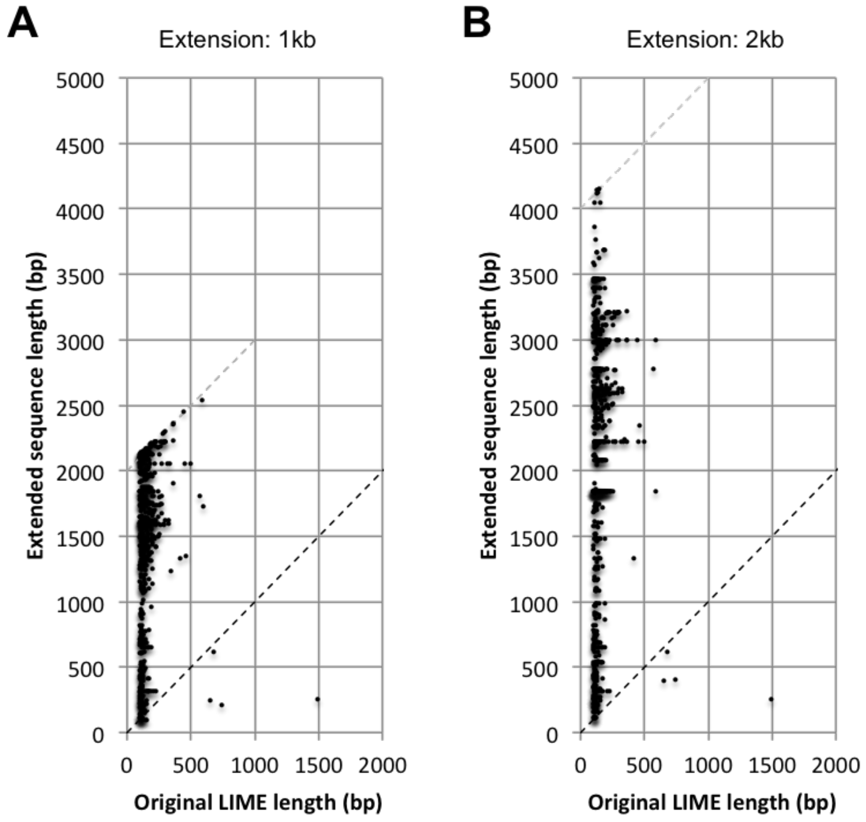

Figure C: Analysis of the output of the genome scanning steps to identify the appropriate thresholds for hit selection. A: number of hits vs the accuracy values, showing linear increase when going down from 1 to 0,94, followed by a sharp increase in the number of hits. B: number of hits vs the length of the hit (as a percentage of the original sequences length), showing that the majority of the hits are greater than 92% of the original sequence length

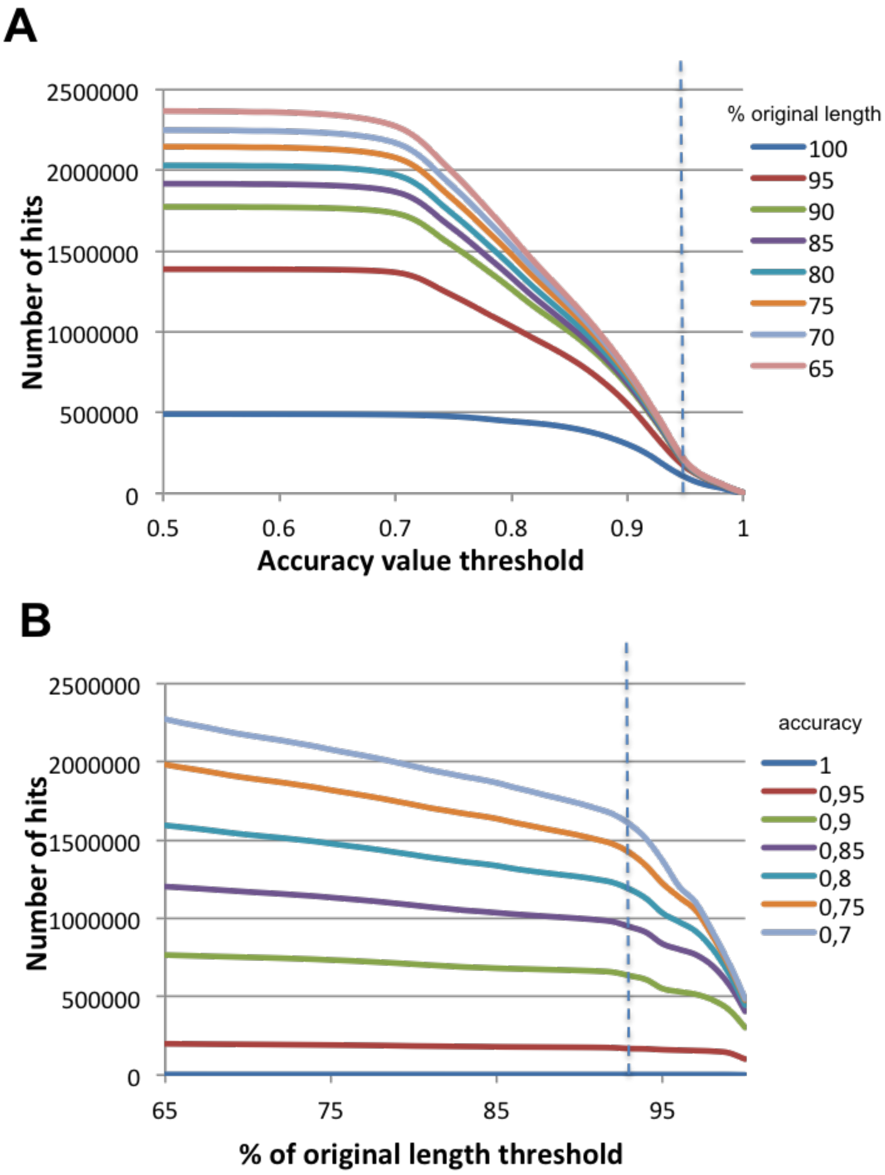

Figure D: Analysis of the number of conserved sequences extracted from the genomes of each of the analysed species. Black: none. Green: between 1 and 10 sequences. Yellow: between 11 and 100 sequences. Red: more than 100 sequences.

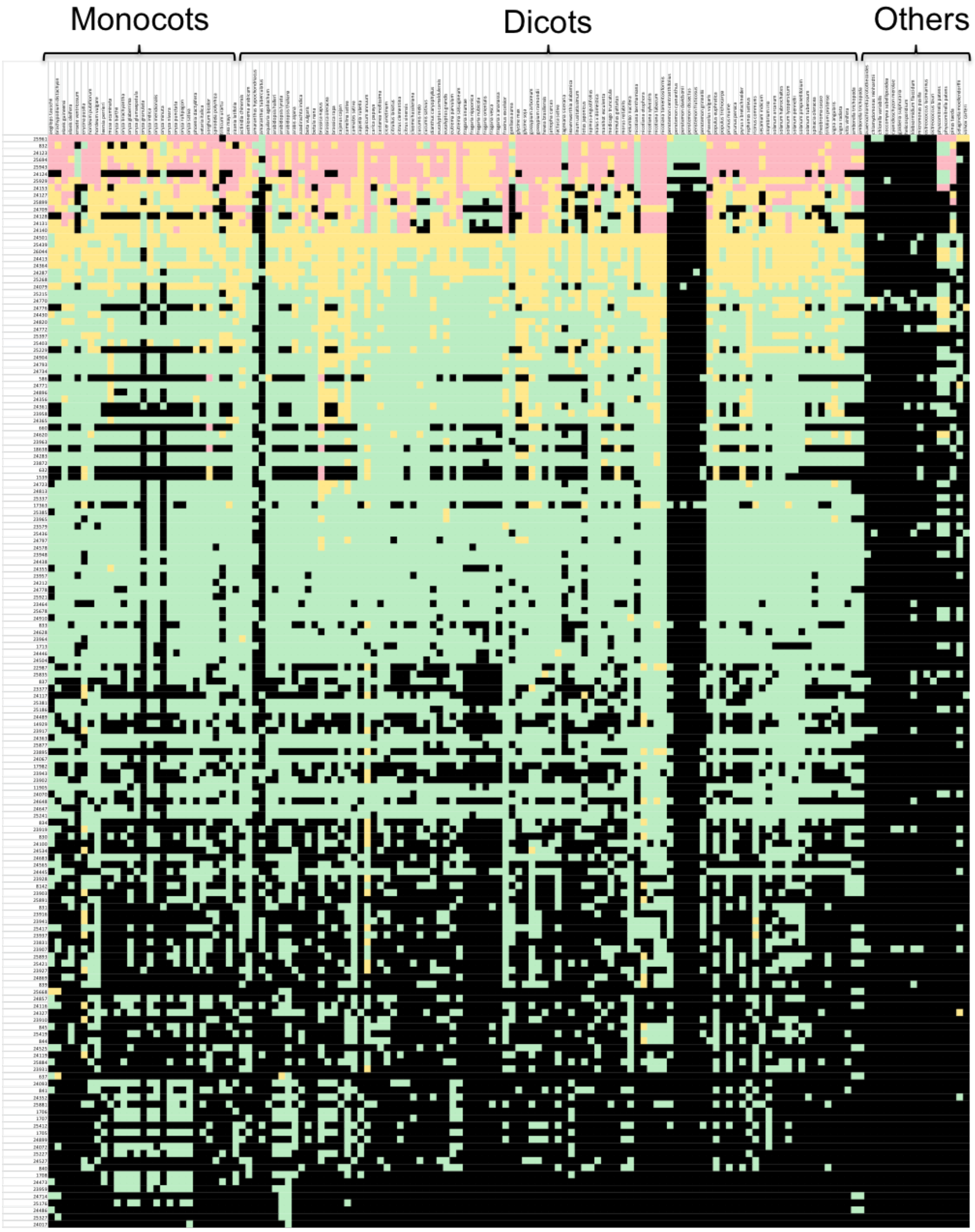

Supplement: S1 Text — This supplementary information includes a detailed description of the bioinformatics pipeline summarized in the text and in Fig 1, as well as the intermediate results obtained with the LIME sequences used as input. (PDF) [file pone.0147692.s001.pdf]
